# CRISPR interference-guided modulation of glucose pathways to boost aconitic acid production in *Escherichia coli*

**DOI:** 10.1186/s12934-020-01435-9

**Published:** 2020-09-03

**Authors:** Qingyang Li, Peng Zhao, Hang Yin, Zhaonan Liu, Haifeng Zhao, Pingfang Tian

**Affiliations:** 1 School of Food Science and Engineering, South China University of Technology, Guangzhou, 510641 China; 2 College of Life Science and Technology, Beijing University of Chemical Technology, Beijing, 100029 China

**Keywords:** Aconitic acid, CRISPR interference, Glucose metabolism, Isocitrate dehydrogenase, Pyruvate kinase

## Abstract

**Background:**

One major mission of microbial breeding is high-level production of desired metabolites. Overproduction of intermediate metabolites in core pathways is challenging as it may impair cell growth and viability.

**Results:**

Here we report that aconitic acid, an intermediate metabolite in tricarboxylic acid (TCA) cycle, can be overproduced by an engineered CRISPR interference (CRISPRi) system in *Escherichia coli*. This CRISPRi system was designed to simultaneously target pyruvate kinase (PK) and isocitrate dehydrogenase (IDH), two enzymes in glycolytic pathway and TCA cycle, respectively. Reverse transcription and quantitative PCR and enzyme activity assays showed that this engineered CRISPRi system significantly repressed the genes encoding IDH and PK, resulting in simultaneous reduction in the activities of IDH and PK. In shake-flask and fed-batch cultivation, this CRISPRi strain produced 60-fold (362.80 ± 22.05 mg/L) and 15-fold (623.80 ± 20.05 mg/L) of aconitic acid relative to the control strain, respectively. In addition, this two-target CRISPRi strain maintained low levels of acetate and lactate, two problematic byproducts.

**Conclusions:**

This work demonstrates that CRISPRi system can improve aconitic acid production by coordinating glycolysis and TCA cycle. This study provides insights for high-level production of the intermediate metabolites in central pathways.

## Background

Aconitic acid was first identified in *Aconitum napellus* and thereby named after this plant. In nature, aconitic acid exists as two isomers (*trans*- and *cis*-) and is one of plentiful organic acids in sugar cane [1]. In addition, aconitic acid is rich in *Pseudomonas* spp. [2] and sugar-containing plants such as wheat (*Triticum aestivum*) [3] and maize (*Zea mays*) [4]. Specially, aconitic acid is an intermediate metabolite in tricarboxylic acid (TCA) cycle and thus is of paramount importance for cell viability. In addition to the participation in core metabolisms and its use as a food additive, *tran*s-aconitic acid has nematicidal [5] and antiedematogenic activities [6], suggesting its potentials in plant protection and therapeutic development. Apart from its versatile bio-functions, aconitic acid can be converted to itaconic acid, which is the feedstock for manufacturing of acrylic plastics, acrylate latexes, super-absorbents, and anti-scaling agents [7]. Aconitic acid can be chemically synthesized through dehydration of citric acid in the presence of concentrated sulfuric acid. However, this leads to the formation of pyrolysates of both citric acid and aconitic acid due to high temperature required in this reaction [8]. Currently, industrial production of aconitic acid relies on the following technical route [9]: propane-1,1,2,3-tetracarboxylic compound is subjected to saponification and dehydrochlorination, and the resulting propylene tetracarboxylate derivative is acidified by sulfuric acid. However, this route brings about troublesome lactones of isocitric acid and alloisocitric acid, which are unwanted byproducts [9], as they not only entangle downstream separation but also increase the production cost of aconitic acid. Hence, it is highly desirable to develop a novel method for the production of aconitic acid. Fortunately, bio-production has emerged as an alternative to conventional chemical synthesis, and it requires moderate instead of stringent reaction conditions.

**Fig. 1:**
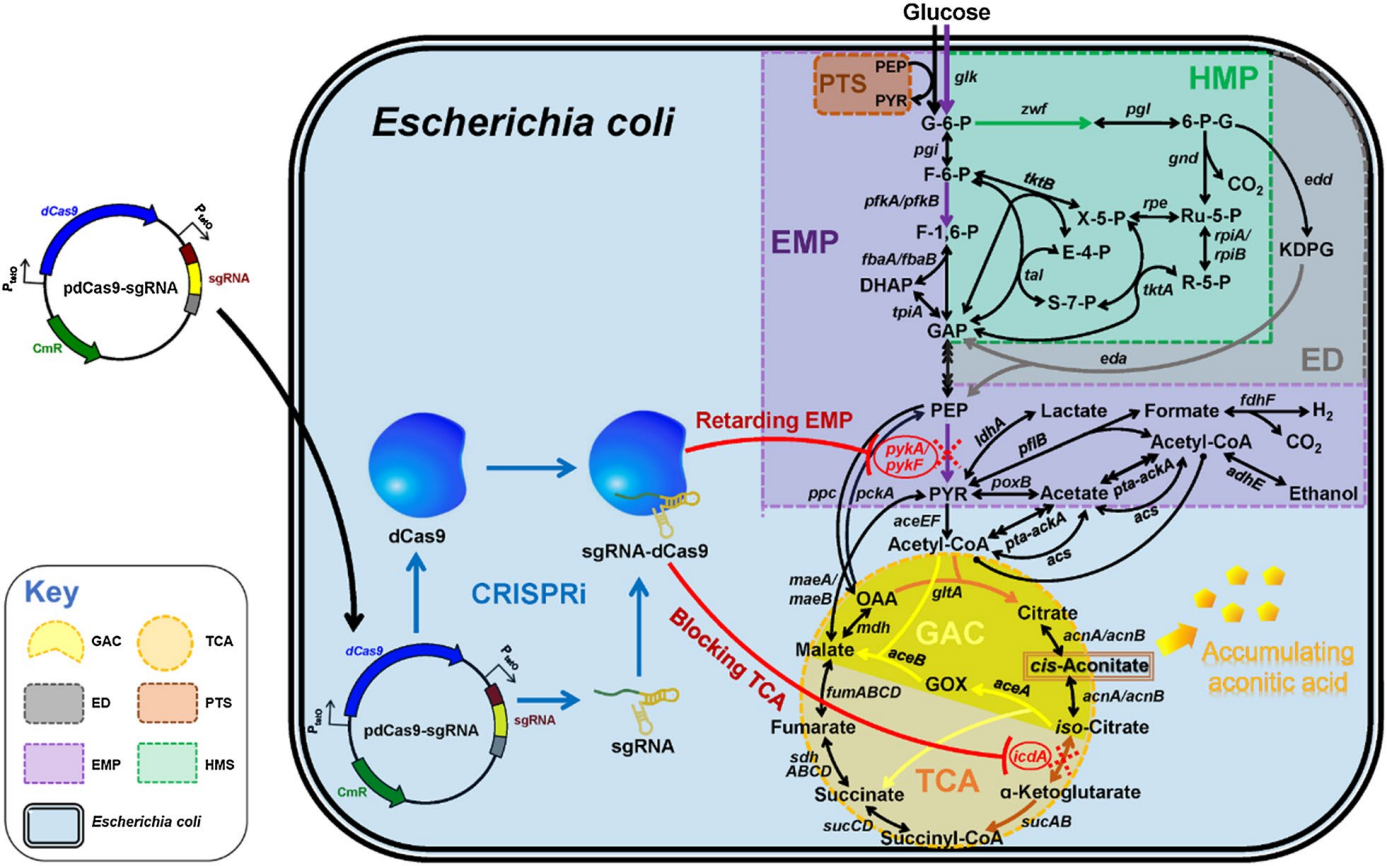
Schematic diagram of improving aconitic acid production by engineering CRISPRi system in *E*. *coli*. Red dashed cross denotes the target enzymes of CRISPRi system. PTS: phosphotransferase system; EMP: Embden-Meyerhof-Parnas pathway; HMP: hexose monophosphate pathway; ED: Entner–Doudoroff pathway; GAC: glyoxylate cycle; TCA: tricarboxylic acid cycle; G-6-P: glucose-6-phosphate; F-6-P: fructose-6-phosphate; F-1,6-P: fructose-1,6-phosphate; 6-P-G: 6-phosphate-gluconolactone; Ru-5-P: ribulose-5-phosphate; R-5-P: ribose-5-phosphate; X-5-P: xylulose-5-phosphate; E-4-P: erythrose-4-phosphate; S-7-P: sedoheptulose-7-phosphate; KDPG: 2-keto-3-deoxy-6-phosphogluconate; GAP: glyceraldehyde-3-phosphate; DHAP: dihydroxyacetone phosphate; PEP: phosphoenolpyruvate; PYR: pyruvate; OAA: oxaloacetate; *glk*: glucokinase coding gene; *pgi*: glucose-6-phosphate isomerase coding gene; *pfkA* and *pfkB*: 6-phosphofructokinase coding genes; *fbaA* and *fbaB*: fructose-bisphosphate aldolase coding genes; *tpiA*: triose-phosphate isomerase coding gene; *zwf*: glucose-6-phosphate dehydrogenase coding gene; *pgl*: 6-phosphogluconolactonase coding gene; *gnd*: 6-phosphogluconate dehydrogenase coding gene; *rpe*: ribulose-phosphate 3-epimerase coding gene; *rpiA* and *rpiB*: ribose-5-phosphate isomerase coding genes; *tktA* and *tktB*: transketolase coding genes; *tal*: transaldolase coding gene; *edd*: 6-phosphogluconate dehydratase coding gene; *eda*: KDPG aldolase coding gene; *pckA*: phosphoenolpyruvate carboxykinase coding gene; *ppc*: phosphoenolpyruvate carboxylase coding gene; *pykA* and *pykF*: pyruvate kinase coding genes; *ldhA*: D-lactate dehydrogenase coding gene; *pflB*: pyruvate formate-lyase coding gene; *poxB*: pyruvate oxidase coding gene; *pta*: phosphate acetyltransferase coding gene; *ackA*: acetate kinase coding gene; *acs*: acetyl-CoA synthetase coding gene; *fdhF*: formate dehydrogenase coding gene; *adhE*: aldehyde-alcohol dehydrogenase coding gene; *aceEF*: pyruvate dehydrogenase-complex coding genes; *maeA* and *maeB*: pyruvic-malic carboxylase coding genes; *gltA*: citrate synthase coding gene; *acnA* and *acnB*: aconitate hydratase coding genes; *icdA*: isocitrate dehydrogenase coding gene; *sucAB*: oxoglutarate dehydrogenase coding genes; *sucCD*: succinyl-CoA synthetase coding genes; *sdhABCD*: succinate dehydrogenase coding genes; *fumA, fumB, fumC* and *fumD*: fumarate hydratase coding genes; *mdh*: malate dehydrogenase coding gene; *aceA*: isocitrate lyase coding gene; *aceB*: malate synthase coding gene

Since aconitic acid is the second metabolite of TCA cycle—the transient intermediate of citric acid to isocitrate reaction [10], it is pretty challenging to accumulate aconitic acid in wild-type *E. coli*. In *E. coli*, most aconitic acid is reversibly converted to citrate and isocitrate by aconitase (ACO, EC 4.2.1.3, encoded by *acnA* and *acnB*) [10] (Fig. 1). Next, isocitrate is converted to alpha-ketoglutarate by isocitrate dehydrogenase (IDH, EC 1.1.1.42, encoded by *icdA* gene), and alpha-ketoglutarate proceeds TCA cycle. Conventional approaches for high-level production of desired metabolites include overexpression of key enzymes and interruption of competing pathways [11]. Following these typical strategies, genetic manipulation of IDH should enable the accumulation of isocitrate and thereby aconitic acid. However, since IDH is a rate-limiting enzyme in TCA cycle, reduction in IDH activity may give rise to imbalance between the low-rate TCA cycle and high-rate glycolysis, thereby leading to buildup of pyruvate and failure to fully enter TCA cycle [12]. As a consequence, excessive pyruvate is converted to acetate, lactate and ethanol [13]. These byproducts not only consume carbon source but also penalize cell viability. Therefore, moderate rather than strong repression of glycolysis allows augmentation of TCA cycle. In view of above information, we anticipate that knocking down rather than knocking out the genes for IDH and PK (EC 2.7.1.40, encoded by *pykA* and *pykF*) might benefit the production of aconitic acid.

To date, scientists have developed a series of strategies for knocking down genes, including antisense RNA (asRNA) technology [14], RNA interference (RNAi) [15], synthetic small-regulatory RNA (sRNAs) [16], Cas13a approach [17] and CRISPR interference (CRISPRi) [18–20]. While asRNA technology involves complicated design of primers [14], RNAi strategy has so far only been applied to down-regulate eukaryotic genes [15], as RNAi machinery has not been found in prokaryotes so far. While sRNAs is time-consuming and sometimes shows low efficiency because its efficiency depends largely on the binding affinity with target mRNA [16]. In recent years, Cas13a is shown to be an RNA- instead of DNA-editing tool [17]. CRISPRi system opens an avenue for simultaneously knockdown multiple genes due to an array of sgRNAs by which dCas9 is directed to desired targets [18–20]. In fact, when dCas9-sgRNA complex acts on a target gene, it blocks RNA polymerases from binding to promoter or open reading frame, thereby impeding transcription initiation or elongation, respectively [21]. This dCas9-based knock-down efficiency can be tuned by varying the target loci and the base pairing between sgRNAs and target genes [21]. That is, CRISPRi can finely tune transcription and thus reconcile cell growth and metabolites production [22]. More critically, CRISPRi system is independent of DNA repair mechanism. In other words, CRISPRi can work in almost all microbes. The aforementioned advantages of CRISPRi system make it an ideal tool to modulate multiple genes in both prokaryotes and eukaryotes.

Given the above information, we conjecture that CRISPRi system might reconcile glycolytic pathway and TCA cycle and thereby improve the production of aconitic acid. To validate this prediction, we engineered CRISPRi systems targeting IDH and PK to divert carbon flux into aconitic acid pathway (Fig. 1). Detailed analysis of cell growth, glucose consumption, gene expression, enzyme activity and metabolic levels aims to systematically assess the effectiveness of CRISPRi system in the repression of the genes for IDH and PK in *E. coli*. Shake-flask and bioreactor cultivation of the recombinant *E. coli* strain harboring CRISPRi system (hereafter CRISPRi strain) were to disentangle the influences of IDH and PK on aconitic acid biosynthesis. Overall, this study aims to enhance the production of aconitic acid by engineering CRISPRi system in *E. coli.*

## Results

### Performance of CRISPRi system

**Fig. 2:**
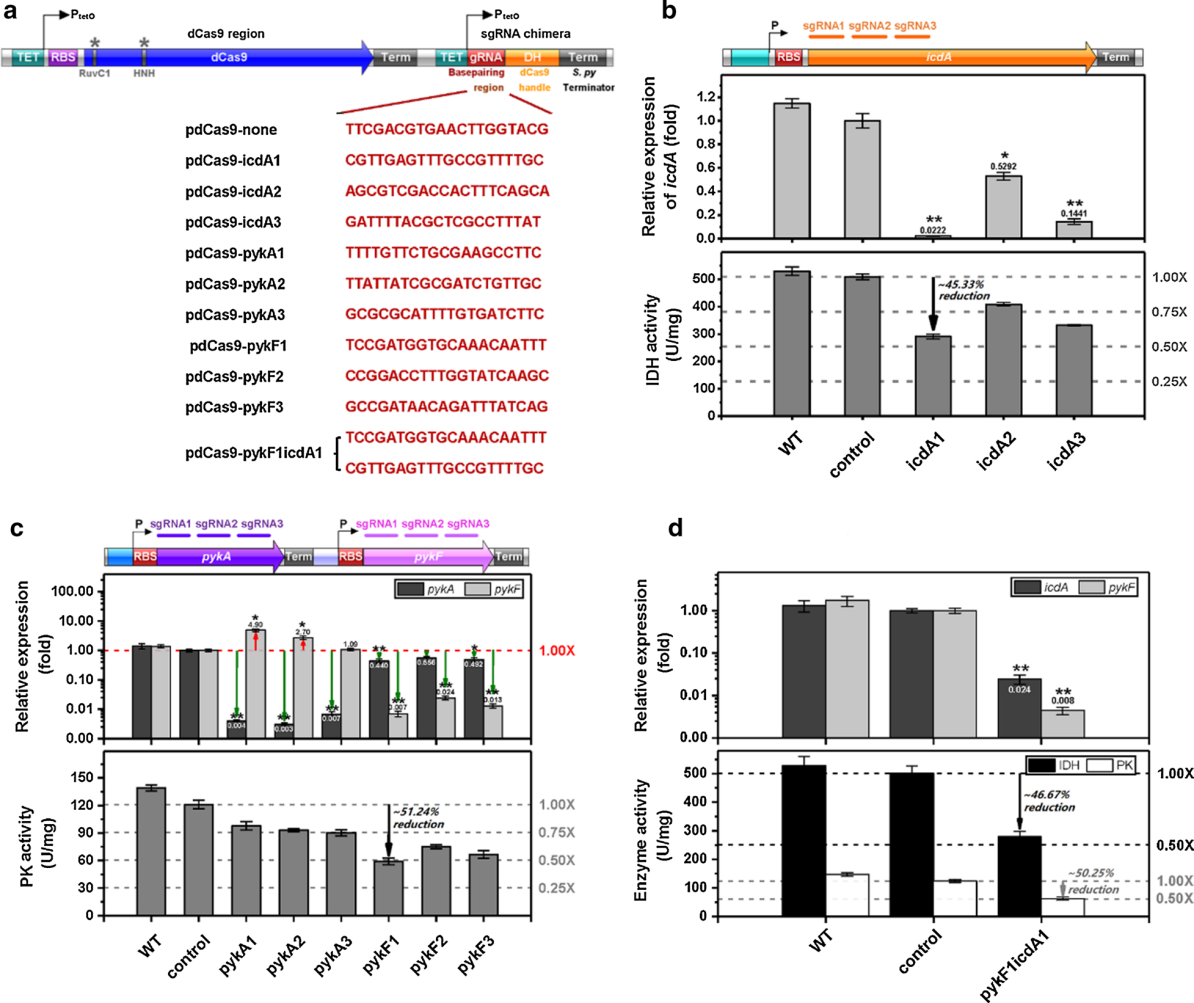
Performance of the CRISPRi system targeting aconitate biosynthesis-related enzymes in glucose pathways. **a** Structural diagram and gRNA sequence of the CRISPRi system. **b** The effect of CRISPRi system targeting *icdA* expression on isocitrate dehydrogenase (IDH) activity in *E. coli*. Relative expression of *icdA* gene in *E. coli* strain harboring CRISPRi system (Top). IDH activity in recombinant *E. coli* (Bottom). **c** Effect of CRISPRi system targeting *pykA/F* on pyruvate kinase (PK) activity in *E. coli*. Relative expression levels of *pykA* and *pykF* genes in *E. coli* strains (Top). PK activity in recombinant *E. coli* (Bottom). **d** Effect of CRISPRi system targeting both *icdA* and *pykF* genes on IDH and PK activities in *E. coli*. Relative expression levels of *icdA* and *pykF* in *E. coli* strains harboring CRISPRi system (Top). Activities of IDH and PK in recombinant *E. coli* (Bottom). IDH: isocitrate dehydrogenase (EC 1.1.1.42, encoded by *icdA* gene); PK: pyruvate kinase (EC 2.7.1.40, encoded by *pykA* and *pykF*). WT: wild-type *E. coli* BL21(DE3) without CRISPRi system; control: recombinant *E. coli* with non-targeting CRISPRi system; icdA1, icdA2 and icdA3: recombinant *E. coli* harboring CRISPRi system targeting different regions of *icdA* gene; pykA1, pykA2 and pykA3: recombinant *E. coli* containing CRISPRi system targeting different regions of *pykA* gene; pykF1, pykF2 and pykF3: recombinant *E. coli* carrying the CRISPRi system targeting various regions of *pykF* gene. Mean ± S.E. (n = 3). *P < 0.05; **P < 0.01

In *E. coli*, biosynthesis of aconitic acid relies on a panel of enzymes. To clarify the influences of their expression on aconitic acid production, three genes *icdA*, *pykA* and *pykF* native to *E. coli* were chosen as the targets of CRISPRi system (Fig. 1). For each gene, three candidate sgRNAs targeting different regions (Fig. 2b, c and Additional file 1: Table S2) were designed and chemically synthesized to construct CRISPRi vectors. The CRISPRi vector with non-targeting sgRNA was used as the control. All CRISPRi vectors were constructed and then individually transformed into competent *E*. *coli* BL21(DE3), resulting in control strain *E. coli* BL21(DE3) + pdCas9-none and three recombinant strains: *E. coli* BL21(DE3) + pdCas9-icdA(1–3), *E. coli* BL21(DE3) + pdCas9-pykA(1–3) and *E. coli* BL21(DE3) + pdCas9-pykF(1–3). Next, reverse transcription and quantitative PCR (RT-qPCR) was performed to decipher the inhibitory efficiency of CRISPRi against *icdA*, *pykA* and *pykF*. The activities of IDH and PK were examined to select best-performing sgRNAs.

Results showed that sgRNAs pykA1, pykA2 and pykA3 suppressed more than 99% of *pykA* expression (Fig. 2c). However, all these transcriptional repression on *pykA* failed to significantly reduce PK activity (Fig. 2c). This result might be explained by that PykA and PykF are isoenzymes, and compared with PykF, PykA contributes less to PK activity [23]. Fortunately, for the CRISPRi strains targeting *icdA* and *pykF*, at least one strain exhibited a marked decrease in the activity of corresponding enzyme (Fig. 2b, c). As shown in Fig. 2b, the sgRNA icdA1 was more powerful than other two sgRNAs in repression of *icdA*, with an inhibitory efficiency of 97.8%, leading to 45.33% reduction in IDH activity. In Fig. 2c, the sgRNA pykF1 outperformed other sgRNAs in repression of *pykF* gene and displayed an inhibitory effect of 99.3%, resulting in 51.24% reduction in PK activity. To simultaneously reduce the activities of IDH and PK, the CRISPRi vector named pdCas9-pykF1icdA1 was tailored by linking two effective sgRNAs (pykF1 and icdA1).

To test the effectiveness of this CRISPRi system, vector pdCas9-pykF1icdA1 was transformed into competent *E*. *coli* BL21(DE3), leading to recombinant strain *E. coli* BL21(DE3) + pdCas9-pykF1icdA1. RT-qPCR and enzyme activity results demonstrated that the CRISPRi vector pdCas9-pykF1icdA1 simultaneously repressed 97.6% of *icdA* expression and 99.2% of *pykF* expression, leading to 46.67% reduction in IDH activity and 50.25% reduction in PK activity (Fig. 2d).

### Shake-flask cultivation of CRISPRi strains

**Fig. 3:**
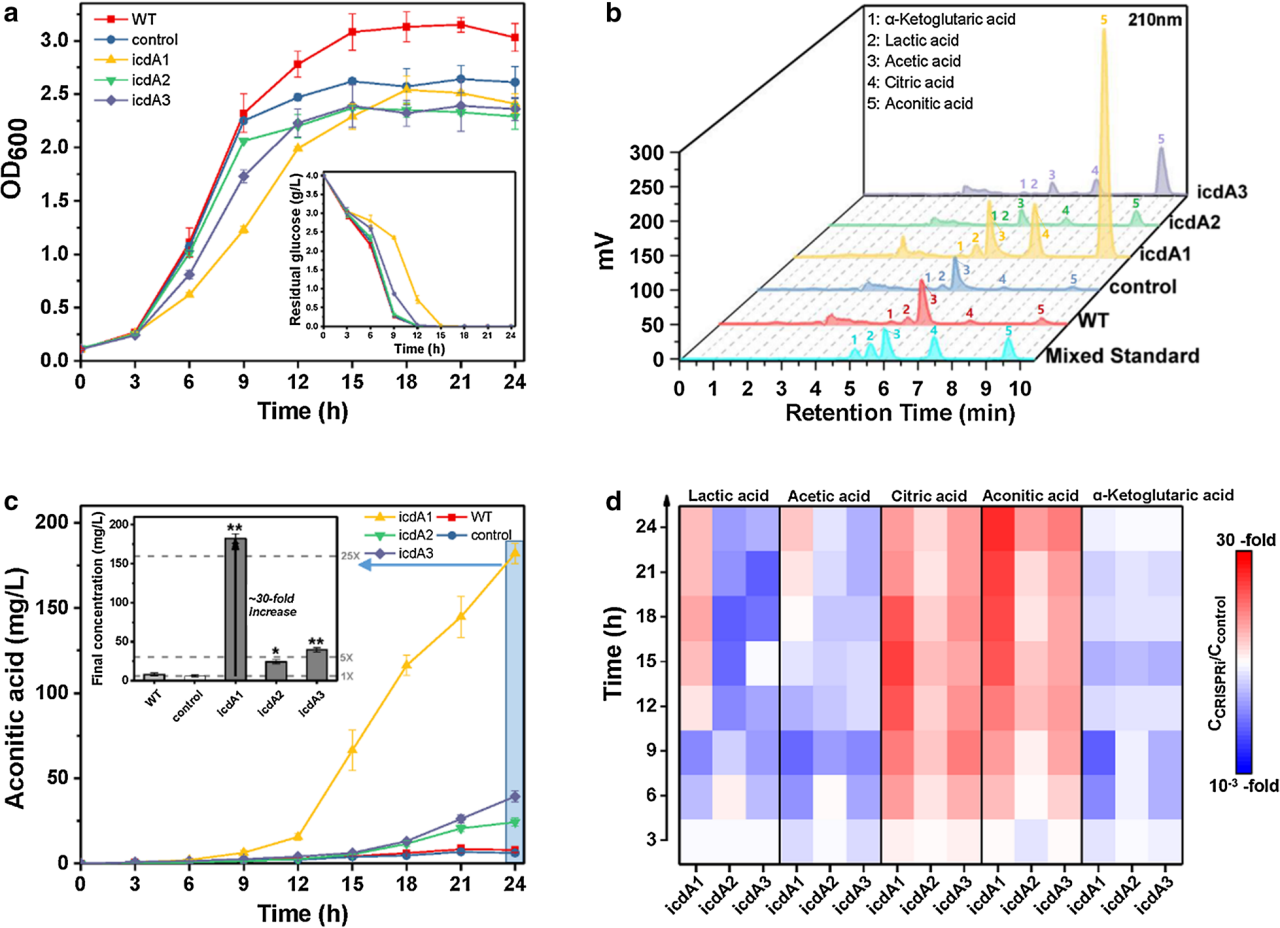
Regulation of the IDH expression of *E. coli* strains in shake-flask cultivation. **a** Growth curve and glucose consumption of wild-type *E. coli* BL21(DE3) and recombinant *E. coli* harboring CRISPRi system. **b** HPLC analysis of mixed standard and the final fermentation broth of *E. coli* strains. **c** Time course of aconitate production and the final concentration of aconitic acid in *E. coli* strains. **d** Relative changes of metabolites in CRISPRi strains (C_CRISPRi_) versus control strain (C_control_). IDH: isocitrate dehydrogenase (EC 1.1.1.42), encoded by *icdA* gene. WT: wild-type *E. coli* BL21(DE3) devoid of CRISPRi system; control: recombinant *E. coli* with non-targeting CRISPRi system; icdA1, icdA2 and icdA3: recombinant *E. coli* harboring the CRISPRi system targeting different regions of *icdA* gene. Mean ± S.E. (n = 3). *P < 0.05; **P < 0.01

To evaluate the performance of CRISPRi strains, we examined their growth rate, glucose consumption and aconitate production in shake-flasks. For CRISPRi system targeting IDH, all three CRISPRi strains targeting different regions of *icdA* presented lower OD_600_ values compared to the control strain in stationary phase (Fig. 3a). Except for the strain employing sgRNA icdA2, other two CRISPRi strains showed retarded growth, and the strain *E. coli* BL21(DE3) + pdCas9-icdA1 demonstrated slowed growth and postponed stationary phase (Fig. 3a). In accordance with cell growth, the glucose consumption of three CRISPRi strains was repressed. As shown in Fig. 3a, while the strain *E. coli* BL21 (DE3) + pdCas9-icdA1 exhausted glucose in 15 h, other two CRISPRi strains and the control strain exhausted it in 12 h (Fig. 3a), 3 h earlier than the strain *E. coli*BL21 (DE3) + pdCas9-icdA1. Of the three CRISPRi strains, the strain *E. coli* BL21(DE3) + pdCas9-icdA1 presented the highest level of aconitic acid (181.02 ± 6.33 mg/L), which was 30 times that of the control strain (6.05 ± 0.55 mg/L) (Fig. 3b, c). To unravel the inhibition of CRISPRi on IDH and central carbon metabolisms, we also examined the byproducts in glucose pathway, including lactate, acetate, citrate and alpha-ketoglutarate. As shown in Fig. 3d, all three CRISPRi strains produced less alpha-ketoglutarate but more citrate compared to the control strain. Compared with the control strain, the strain *E. coli* BL21(DE3) + pdCas9-icdA1 produced more lactate in log phase and more acetate in stationary phase (Fig. 3d). The above results suggested that only inhibition of IDH is sufficient to retard glucose consumption and cell growth, leading to buildup of the metabolites upstream alpha-ketoglutarate in TCA cycle. However, excessive inhibition of IDH caused retarded aerobic metabolism and buildup of anaerobic metabolites in Embden-Meyerhof-Parnas (EMP) pathway.

**Fig. 4:**
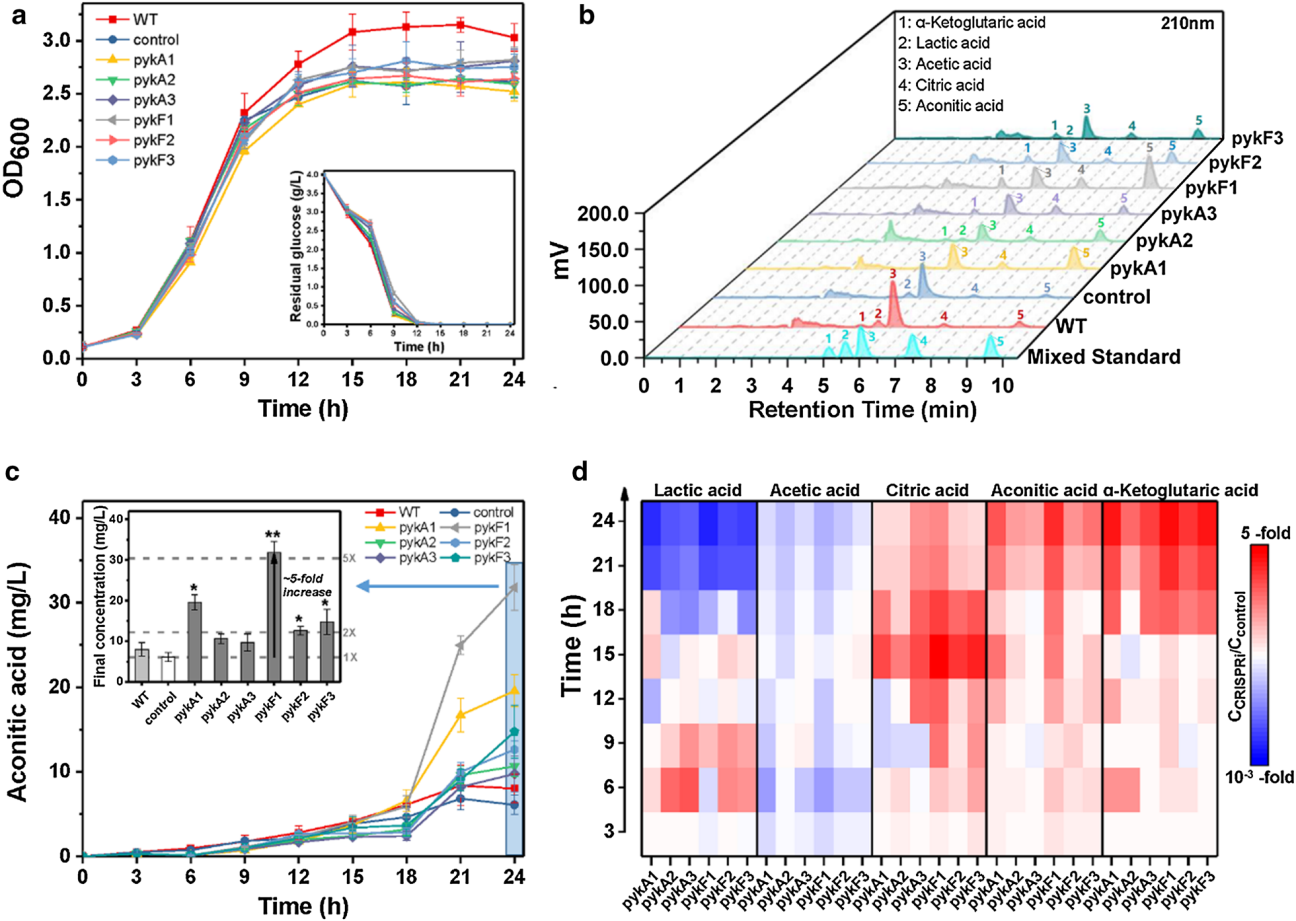
Regulation of the PK expression of *E. coli* strains in shake-flask cultivation. **a** Growth curve and glucose consumption of wild-type *E. coli* BL21(DE3) and recombinant *E. coli* strains harboring CRISPRi system. **b** HPLC analysis of mixed standard and the final fermentation broth of *E. coli* strains. **c** Time course of aconitate production and the final concentration of aconitic acid in *E. coli* strains. **d** Relative changes of metabolites in CRISPRi strains (C_CRISPRi_) versus control strain (C_control_). PK: pyruvate kinase (EC 2.7.1.40), encoded by *pykA* and *pykF*. WT: wild-type *E. coli* BL21(DE3) without CRISPRi system; control: recombinant *E. coli* with non-targeting CRISPRi system; pykA1, pykA2 and pykA3: recombinant *E. coli* containing CRISPRi system targeting different regions of *pykA* gene; pykF1, pykF2 and pykF3: recombinant *E. coli* carrying CRISPRi system targeting various regions of *pykF* gene. Mean ± S.E. (n = 3). *P < 0.05; **P < 0.01

With respect to the CRISPRi system targeting PK, all six CRISPRi strains targeting different regions of *pykA* or *pykF* displayed similar growth with the control strain (Fig. 4a). Consistent with cell growth, no significant difference was observed in the glucose consumption of all above strains (Fig. 4a). After 24 h aerobic growth, the strain *E. coli* BL21(DE3) + pdCas9-pykF1 produced more aconitic acid compared with other five CRISPRi strains, as its final titer reached up to 31.79 ± 2.72 mg/L, which was 5-fold increase relative to the control strain (Fig. 4b, c). All six CRISPRi strains generated more citrate and alpha-ketoglutarate relative to the control strain (Fig. 4d). In addition, these CRISPRi strains synthesized less lactate and acetate relative to the control strain (Fig. 4d). Collectively, CRISPRi-dependent inhibition of PK did not substantially constrain glucose consumption and cell growth. Instead, it facilitated the accumulation of intermediate metabolites in TCA cycle. Furthermore, CRISPRi-based inhibition of PK effectively reduced the metabolites using pyruvate as precursors in glycolytic pathway.

**Fig. 5:**
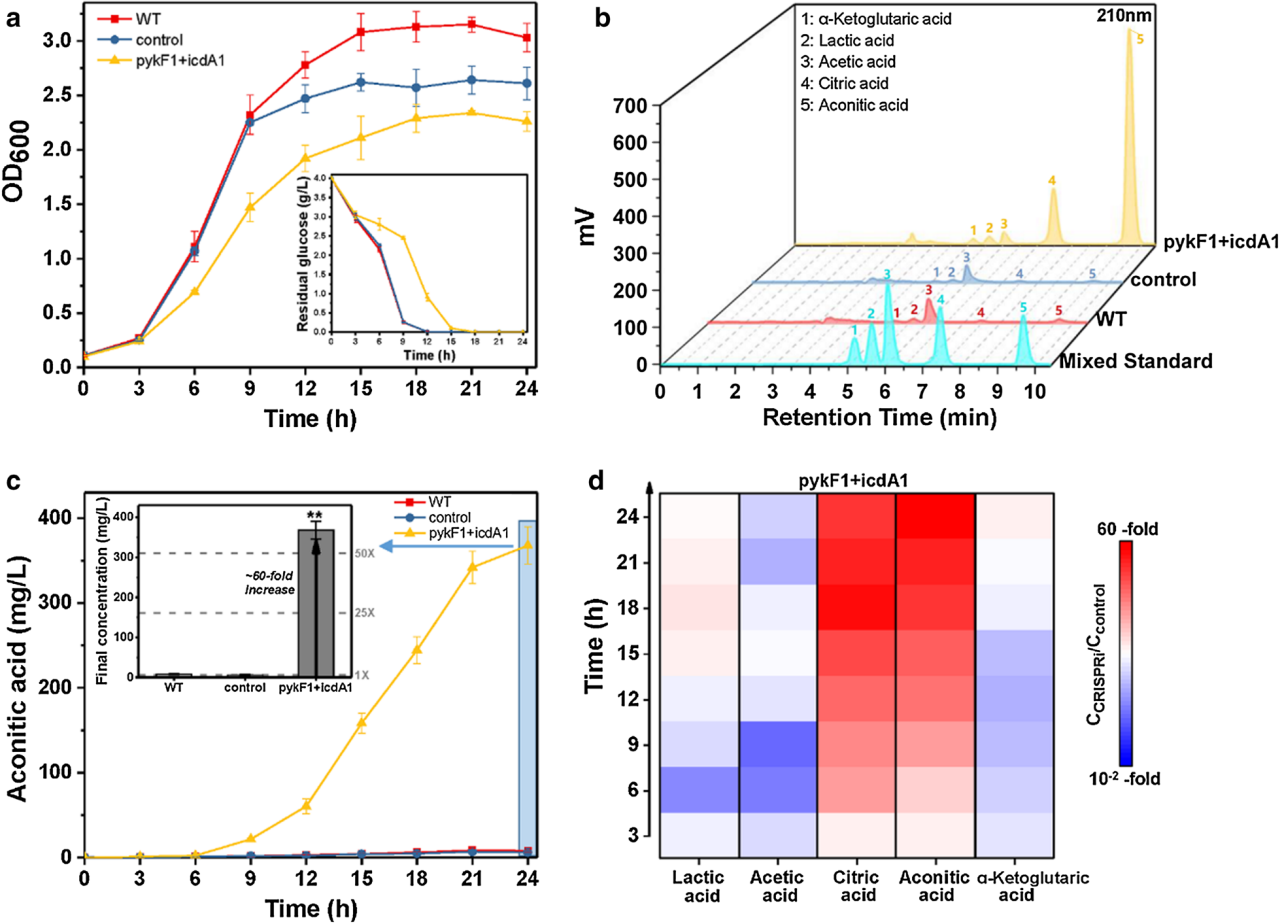
Simultaneous regulation of IDH and PK of *E. coli* strains in shake-flask cultivation. **a** Growth curve and glucose consumption of wild-type *E. coli* BL21(DE3) and recombinant *E. coli* harboring CRISPRi system. **b** HPLC analysis of mixed standard and the final fermentation broth of *E. coli* strains. **c** Time course of aconitic acid production and its final concentration in *E. coli* strains. **d** Relative changes of metabolites in strain *E. coli* BL21(DE3) + pdCas9-pykF1icdA1 (C_CRISPRi_) versus control strain (C_control_). IDH: isocitrate dehydrogenase (EC 1.1.1.42), encoded by *icdA* gene; PK: pyruvate kinase (EC 2.7.1.40), encoded by *pykA* and *pykF*. WT: wild-type *E. coli* BL21(DE3) without CRISPRi system; control: recombinant *E. coli* with non-targeting CRISPRi system; pykF1 + icdA1: recombinant *E. coli* carrying CRISPRi system simultaneously targeting *pykF* and *icdA* genes. Mean ± S.E. (n = 3). **P < 0.01

For the CRISPRi system targeting both IDH and PK, the corresponding recombinant CRISPRi strain simultaneously targeting *pykF* and *icdA* displayed slower growth compared with the CRISPRi strains only targeting *pykF* or *icdA* (Figs. 3a, 4a and 5a). In accordance with cell growth, the strain *E. coli* BL21(DE3) + pdCas9-pykF1icdA1 consumed the least glucose among all *E. coli* strains (Figs. 3a, 4a and 5a). After 24 h shake-flask cultivation, the strain *E. coli* BL21(DE3) + pdCas9-pykF1icdA1 produced more aconitic acid compared to other recombinant and wild-type *E. coli* (Figs. 3c, 4c and 5c), and the final titer (362.80 ± 22.05 mg/L) was approximately 60 times that of the control strain (Fig. 5b, c). Moreover, the strain *E. coli* BL21(DE3) + pdCas9-pykF1icdA1 generated more citrate but less alpha-ketoglutarate, acetate and lactate compared to the control strain (Fig. 5d). Overall, simultaneous inhibition on PK and IDH not only boosted the accumulation of intermediates in aconitate pathway but also reduced the formation of byproducts using pyruvate as the precursor.

### Fed-batch cultivation of CRISPRi strains

**Fig. 6:**
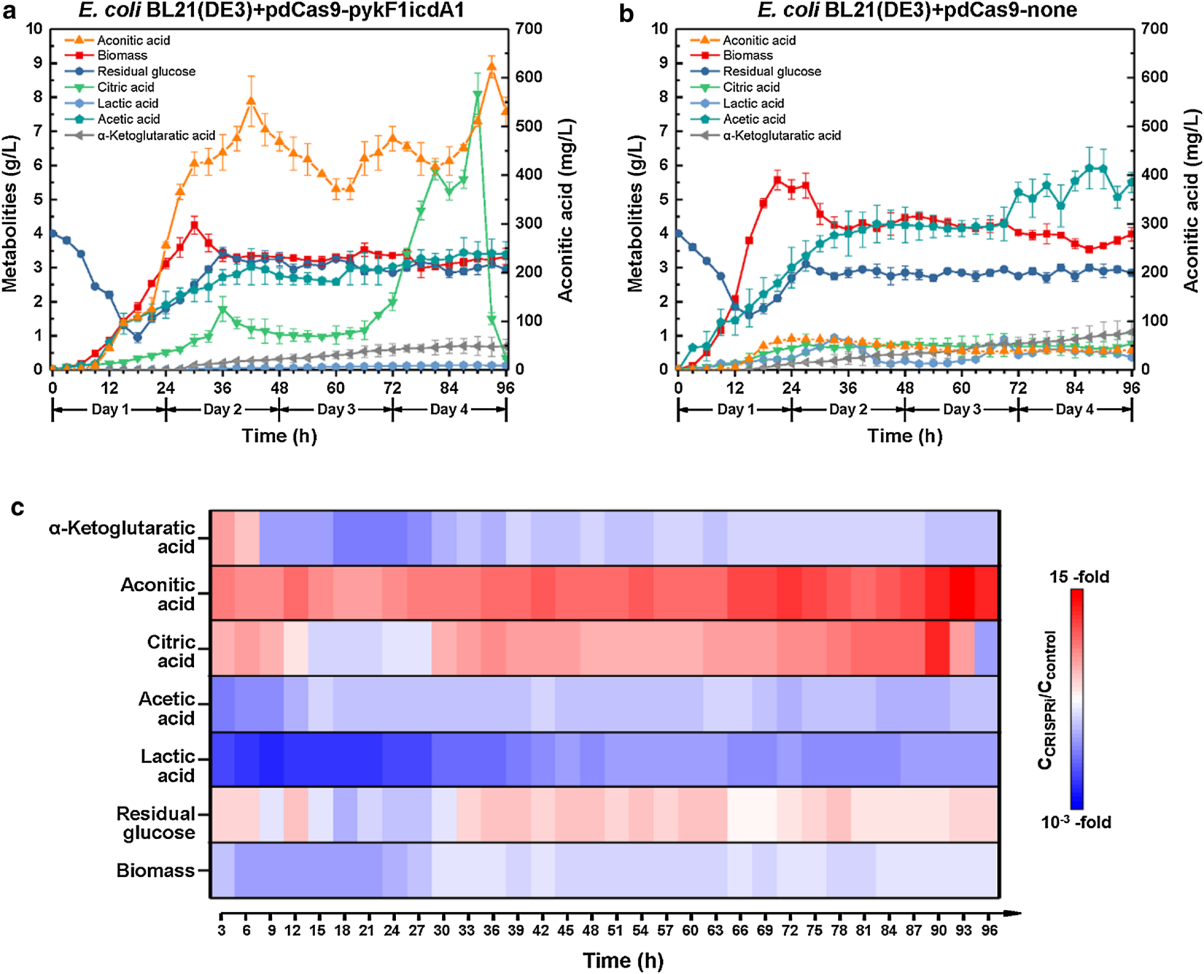
Bioreactor cultivation of the recombinant *E. coli* strains harboring CRISPRi system. **a** Fed-batch cultivation of the control strain *E. coli* BL21(DE3) carrying non-targeting CRISPRi vector pdCas9-none. **b** Fed-batch cultivation of the recombinant *E. coli* harboring CRISPRi vector pdCas9-pykF1icdA1 simultaneously targeting *pykF* and *icdA* genes. **c** Ratio variation in metabolites of the strain *E. coli* BL21(DE3) + pdCas9-pykF1icdA1 (C_CRISPRi_) versus control strain (C_control_) over time. Mean ± S.E. (n = 3)

The recombinant CRISPRi strain targeting both PK and IDH produced more aconitic acid in shake-flask relative to other *E. coli* strains (Figs. 3c, 4c and 5c). To further elucidate the performance of CRISPRi system, the strain *E. coli* BL21(DE3) + pdCas9-pykF1icdA1 and the control strain were independently cultivated in a 5 L bioreactor. Results showed that the strain *E. coli* BL21(DE3) + pdCas9-pykF1icdA1 manifested aconitic acid peak (623.80 ± 20.05 mg/L) at 93 h (Fig. 6a). This concentration was approximately 15 times that of the control strain (Fig. 6c). In addition, this strain manifested citric acid peak (569.33 ± 40.67 mg/L) at 90 h (Fig. 6a). Strikingly, this citric acid peak declined dramatically during 90 h-96 h (Fig. 6aa). Unlike the strain *E. coli* BL21(DE3) + pdCas9-pykF1icdA1, the control strain produced much less citrate (Fig. 6b). As for byproducts, compared with the control strain, the strain *E. coli* BL21(DE3) + pdCas9-pykF1icdA1 produced less lactic acid and acetic acid during the entire fermentation (Fig. 6c). Notably, the inhibition of CRISPRi on *pykF* and *icdA* genes caused simultaneous reduction in the activities of PK and IDH, which exerted a burden on cell growth. As shown in Fig. 6a, b, the highest biomass of *E. coli* BL21(DE3) + pdCas9-pykF1icdA1 was only 4.22 ± 0.22 g/L, whilst the maximum biomass of the control strain was 5.56 ± 0.29 g/L.

## Discussion

In view of Fig. 1, high-level production of aconitic acid necessitates intensification of TCA cycle and simultaneous attenuation of alpha-ketoglutarate synthesis. However, this is extremely challenging because TCA cycle is closely coupled with cell viability, and no matter intensification or attenuation of TCA cycle in most cases may compromise cell growth and viability [24]. Hence, in present study, CRISPRi rather than CRISPR editing was applied to reconcile cell growth and aconitic acid biosynthesis, as CRISPRi is not lethal to cells in most cases. Unlike RNAi that acts on mRNA via a double-stranded RNA-induced silencing complex which recognizes and degrades the corresponding mRNA [15], CRISPRi acts on DNA through a dCas9-sgRNA complex which prevents RNA polymerase from binding to DNA coding strand [21]. Clearly, RNAi suppresses gene expression at posttranscriptional level, whilst CRISPRi does this at transcriptional level (prior to mRNA formation). Moreover, while CRISPRi system can work in both prokaryotes and eukaryotes, RNAi machinery is mainly applicable in eukaryotes, as RNAi mechanism has not been identified in prokaryotes, and development of RNAi machinery in prokaryotes remains a challenge. These advantages of CRISPRi makes it a promising tool for coordinating cell growth and aconitic acid production.

To boost aconitic acid production, IDH and PK were chosen as the targets of CRISPRi system. Although the engineered CRISPRi system exhibited a strong repression on the expression of *icdA*, *pykA* and *pykF* genes, with inhibitory efficiency of 97–99%, the activities of IDH and PK were repressed by at most 50% (Fig. 2). This phenomenon might be explained by that mRNA abundance cannot precisely embody enzyme activity. This opinion is supported by the study of Sinskey group, which pointed out not only the disparity between mRNA abundance and enzyme activity but also the virtual impossibility to generally predict protein activity from quantitative transcriptome data [25]. Additionally, enzyme activity only reflects the conversion of substrate to product, and there exist multiple pathways towards substrate and product. For instance, there are at least two pathways towards D-lactic acid [20]. Another finding of this study is that 97.6% inhibition of *pykF* is more effective than 99.7% inhibition of *pykA* in reducing PK activity (Fig. 2C), indicating that compared with PykA, PykF exhibits better catalytic activity against substrate, and this viewpoint is supported by prior studies [23, 26]. It seems that PykF is more sensitive to environmental changes compared with PykA, and the coexistence of PykF and PykA represents an exquisite mechanism enabling *E*. *coli* to cope with a wide range of environmental changes. As for CRISPRi targeting IDH, although no more than 50% of IDH activity was repressed, in shake-flask cultivation, the corresponding CRISPRi strain showed improved production of aconitic acid and byproducts such as lactate, acetate and citrate (Fig. 3), indicating that this CRISPRi system attenuated the formation of alpha-ketoglutarate and thereby facilitated the accumulation of the substrates upstream alpha-ketoglutarate. However, there existed a threshold of CRISPRi inhibition against *icdA* expression, and above this threshold TCA cycle would be hindered and pyruvate-derived byproducts would be generated. For the CRISPRi system only targeting PK, the corresponding CRISPRi strain presented significantly reduced lactate and acetate but slightly enhanced aconitic acid, citrate and alpha-ketoglutarate (Fig. 4). These results indicated that strongly repressing the expression *of pykA* or *pykF* reduced glycolysis and in turn attenuated the metabolic flux towards pyruvate. As a result, carbon flux was diverted into TCA cycle. For the CRISPRi strain targeting both IDH and PK, it showed not only remarkably improved aconitic acid and citrate but also reduced the levels of acetate, lactate and alpha-ketoglutarate (Fig. 5). This phenomenon indicated that simultaneous repression of the expression of *icdA* and *pykF* benefits the conversion of pyruvate to citrate and subsequent aconitic acid. Overall, CRISPRi system can coordinate EMP and TCA cycle and thus reasonably allocate cellular resources.

Apart from coordination of glycolysis and TCA cycle, active *E. coli* growth is also crucial for aconitic acid production. In general, plasmids halt microbial growth. As shown in Figs. 3a, 4a and 5a, the CRISPRi strains presented lower OD_600_ values in stationary phase compared to wild-type *E*. *coli*, indicating that CRISPRi system imposed a burden on *E*. *coli* growth. This can also be evidenced by the result of bioreactor cultivation. As shown in Fig. 6a, the CRISPRi strain targeting both IDH and PK displayed retarded growth, and aconitic acid ceased to accumulate during the late stage of fermentation. In fact, this could be ascribed to the following reasons: (i) Suppressing multiple essential genes might cause retarded growth [27], and active growth is a prerequisite for the production of aconitic acid. (ii) Under acidic conditions, *cis*-aconitic acid was promptly converted to thermodynamically stable *trans*-aconitic acid [28] which inhibited aconitase [29] and fumarase [30]. The *trans*-aconitic acid blocked the formation of aconitic acid and in turn impeded aerobic metabolism. (iii) Citrate is an allosteric inhibitor of 6-phosphofructokinase-1 (PFK, EC 2.7.1.11, encoded by *pfkA*) (Fig. 1) which is a rate-liming enzyme of glycolysis. Thus, during the late stage of fermentation, the increasing citric acid hijacked the metabolic flux towards EMP pathway [31] (Fig. 6a), leading to diminished glucose uptake, halted growth and reduced metabolites. The high level of citric acid in the medium can be reused by *E*. *coli* upon activation or incorporation of citrate uptake system and citrate lyse [32]. It was reported that CitS protein is a Na^+^-dependent citrate carrier from *Klebsiella pneumoniae* [33]. Hence, incorporation of *citS* gene into CRISPRi strain may allow citrate utilization. As for the sudden decline of citric acid since 90 h, it may be attributed to the activation of uptake system or conversion to other metabolites.

Timely removal of stress and improvement of tolerance are crucial for high-level production of desired metabolites [34]. To this end, scientists have developed a series of strategies, including global transcription machinery engineering [35], metabolite-responsive dynamic control [36], quorum sensing (QS)-based dynamic regulation [37], transporter engineering [38], subcellular compartmentalization of biosynthesis pathways [39], and CRISPRi-based modulation of transcriptional factors [40]. These strategies enable ordinated cell growth and metabolites formation. For instance, one group from Massachusetts Institute of Technology constructed an EsaI/EsaR QS system-based knock-down circuit to optimize the production of myoinositol, glucaric acid and shikimic acid [37]. In addition to above strategies, in present study, CRISPRi system could be integrated into *E*. *coli* genome to alleviate plasmid burden. CRISPRi can also be linked to QS, and the resulting module can dynamically modulate gene expression [41]. Apart from the versatile CRISPR tools [17, 21], one promising strategy capable of coordinating cell growth and metabolites formation may be orthogonal expression system which allows decoupling of aconitic acid biosynthesis from TCA cycle. This hierarchical expression system comprises mainly orthogonal ribosomes [42] and XNA polymerase [43]. Recently, an orthogonal ribosome system has been exploited to decouple plasmid-based gene expression from host metabolisms [44]. In this study, two sets of ribosomes fulfill distinct tasks, leading to improved adaptability between plasmids and host cell [44]. Clearly, this xenobiology-based orthogonal expression system opens new avenues for gene regulation and decoupling of biochemical events. Despite the feasibility of state-of-the-art approaches, CRISPRi remains popular in global and local gene regulation due mainly to the flexible sgRNAs which direct dCas9 to target multiple chromosomal sites [21]. Overall, this study provides valuable insights for overproduction of aconitic acid and other intermediate metabolites in core pathways.

## Conclusions

In summary, this is the first report of engineering CRISPRi system to improve aconitic acid production, and the behind mechanism is coordination of glycolysis and TCA cycle. In shake-flask and fed-batch cultivation, the CRISPRi strain targeting both *pykF* and *icdA* produced 60-fold (362.80 ± 22.05 mg/L) and 15-fold (623.80 ± 20.05 mg/L) of aconitic acid compared with the control strain, respectively. During entire fermentation, this two-target CRISPRi strain presented low levels of acetate and lactate, two problematic byproducts. This work provides insights for overproduction of aconitic acid and other intermediate metabolites in core pathways.

## Materials and methods

### Strains, medium and chemicals

The strains and vectors used in this study are listed in Additional file 2: Table S1. Strains of *E. coli* BL21(DE3) and *E. coli* Top10 were purchased from Biomed Co., Ltd. *E. coli* BL21(DE3) was used as the host strain of CRISPRi system, and *E. coli* Top10 was employed for vector construction. For vector construction, all strains were grown in LB medium containing 10 g/L tryptone, 5 g/L yeast extract, 10 g/L NaCl, and 25 mg/L chloramphenicol (CM). In CRISPRi experiments, strains were grown in M9 medium containing 12.8 g/L Na_2_HPO_4_·7H_2_O, 3 g/L KH_2_PO_4_, 0.5 g/L NaCl, 1 g/L NH_4_Cl, 0.5 g/L MgSO_4_·7H_2_O, 11.1 mg/L CaCl_2_, 4 g/L glucose, 25 mg/L CM and 1 μM anhydrotetracycline (aTc). The aTc concentration was based on pre-experiments (Additional file 3: Fig. S1). Taq plus DNA polymerase, restriction enzymes, and T4 DNA ligase were purchased from TaKaRa (Dalian, China). Primer synthesis and DNA sequencing were accomplished by Biomed Co., Ltd. Other chemicals for gel electrophoresis and HPLC analysis were purchased from Sigma-Aldrich (Shanghai, China).

### Construction of recombinants

To regulate glucose pathways for aconitic acid production, two rate-limiting enzymes IDH and PK were chosen as the targets of CRISPRi system. The CRISPRi vectors targeting IDH and PK were derived from vector plv-dCas9-sgRNA [45]. The vector plv-dCas9-sgRNA contains an inactive *dCas9* from *Streptococcus pyogenes* and a sgRNA chimera, both are expressed under TetR-inducible P_tetO_ promoter. The sgRNA chimera contains three parts: a 20 bp DNA complementary to the target sequence called base-pairing region (BPR), a 42 bp hairpin region for dCas9 binding termed dCas9 handle (DH), and a 40 bp terminator named rrnB (Ter) (Fig. 2a). The *Bsp*Q I sites in vector plv-dCas9-sgRNA were used for directional cloning of any sgRNA into this vector without leaving a scar. To construct CRISPRi vectors, only the sgRNA sequence in vector plv-dCas9-sgRNA needs to be replaced. Briefly, two complementary oligonucleotides containing 20 bases homologous to the target sequence plus 3 bases at the 5′ end of each oligonucleotide matching the *Bsp*Q I-digested vector were synthesized, annealed, phosphorylated and cloned into plv-dCas9-sgRNA. Subsequent ligation resulted in desired CRISPRi vectors.

To ensure efficient inhibition, three candidate sgRNAs targeting the different regions of each aconitate biosynthesis-related genes were chemically synthesized (Additional file 1: Table S2), and the resulting CRISPRi vectors were named after respective genes (Additional file 2: Table S1). That is, vectors ‘pdCas9-icdA1’, ‘pdCas9-icdA2’ and ‘pdCas9-icdA3’ denote the CRISPRi vectors targeting the different regions of the IDH coding gene *icdA*; ‘pdCas9-pykA1’, ‘pdCas9-pykA2’ and ‘pdCas9-pykA3’ stand for the CRISPRi vectors targeting the different regions of PK coding gene *pykA*; ‘pdCas9-pykF1’, ‘pdCas9-pykF2’ and ‘pdCas9-pykF3’ indicate the CRISPRi vectors targeting the PK coding gene *pykF*, and ‘pdCas9-pykF1icdA1’ refers to the CRISPRi vector simultaneously targeting two genes *pykF* and *icdA* (Fig. 2b and Table S1). In addition, the vector ‘pdCas9-none’ harboring non-targeting sgRNA was used as the control. Subsequently, all above vectors were individually transformed into competent *E*. *coli* cells and confirmed by colony PCR and DNA sequencing.

### Transformation and screening

100 μl competent *E. coil* cells were mixed with 100 ng vector in an Eppendorf tube, and incubated on ice for 30 min. Next, the mixture was heated to 42 °C in a water bath for 90 s and then suddenly cooled on ice for 2 min. After transformation, the mixture with 900 μl SOC medium was incubated in a rotatory shaker at 180 rpm and 37 °C. After 1 h recovery, positive clones were screened by LB plates (LB medium with 1.5% agar) containing 25 µg/mL CM and at 37 °C.

### Reverse transcription and quantitative PCR (RT-qPCR)

Wild-type *E. coli* BL21(DE3) and all recombinant *E. coli* strains were grown in 37 °C for 8 h, and then harvested by centrifugation at 12,000 rpm and 4 °C. The *E. coli* cells were immediately chilled with liquid nitrogen to avoid RNA degradation, and then subjected to RNA extraction using RNAiso Plus (Takara, Dalian, China). Absorbance values at 260 and 280 nm were measured by a Nanodrop instrument to determine the quantity and purity of RNA. RNA samples were used to synthesize cDNA through reverse transcription (RT) using PrimeScript™ RT reagent Kit (Takara, Dalian, China). Quantitative PCR (qPCR) of cDNA was performed on Applied Biosystems 7300 Real-Time PCR System with Relative Expression Software Tool 2009 v2.0.13 using SYBR^®^ Premix Ex Taq™ II (Takara, Dalian, China). The qPCR of RNA samples without RT was conducted to exclude the effects of genomic DNA contamination. The primers for RT-qPCR analysis were designed using Primer Premier 5.0 software to generate amplicons of 90–110 nt (Additional file 1: Table S2). Amplification efficiency of all primer pairs needs to be higher than 99% based on the slope of a standard curve of serial dilutions of cDNA. Data of RT-qPCR were analyzed using 2^−∆∆Ct^ strategy with *E. coli* 16S rRNA as an internal standard. All samples were performed in triplicate.

### Enzyme activity assay

Wild-type *E. coli* BL21(DE3) and all recombinant *E. coli* were cultivated in 37 °C till stationary phase, and then collected by centrifugation at 10,000 rpm for 10 min. The harvested cells were resuspended in 5 mL PBS (pH 7.3). 10 μL of β-mercaptoethanol (10 mM) was added to repress protease activity. Next, *E. coli* cells were sonicated in an ice bath and centrifuged at 12,000 rpm for 5 min to obtain supernatant. To determine IDH activity, 100 μL supernatant was added to 1.9 mL reaction system containing Tris–HCl (20 mM, pH 8.0), MgCl_2_ (2 mM), DL-isocitric acid trisodium (5 mM) and NAD^+^ (2 mM), and incubated at 37 °C for 5 min. Subsequently, the increase in absorbance of NADH at 340 nm was detected by the Cary 300 Bio UV–visible spectrophotometer (Varian Medical Systems Inc., U.S.). One unit (U) of IDH activity was defined as the amount of IDH that produces 1 μM NADH per minute. In addition, PK activity was detected by the PK Test Kit (Jiancheng Bioengineering Institute, Nanjing). One unit (U) of PK activity was defined as the amount of PK that converts 1 μmol of phosphoenolpyruvate (PEP) to pyruvate per minute. Protein concentration was measured using the Quick Start Bradford Protein Assay Kit (Bio-Rad, U.S.), and bovine serum albumin (BSA) was employed as the standard protein. Determination of protein concentration followed the kit instructions and the Bradford method [46].

### Shake-flask cultivation of CRISPRi strains for production of aconitic acid

The CRISPRi vectors targeting one or two rate-limiting enzymes that affect aconitic acid biosynthesis were transformed into *E. coli* BL21(DE3). The strain harboring vector pdCas9-none was used as a control (Additional file 2: Table S1). All above recombinant *E. coli* strains and wild-type *E. coli* BL21(DE3) were grown in LB medium for 16 h and subsequently transferred to shake flasks containing M9 medium and 25 mg/L CM. These strains were cultivated in a shaker at 150 rpm and 37 °C. After 3 h cultivation, aTc at final concentrations of 1 µM was added to induce dCas9 expression. The fermentation broth was sampled every 3 h to examine cell growth, glucose consumption and metabolites formation.

### Bioreactor cultivation of *E. coli* for aconitic acid biosynthesis

The CRISPRi strains and control strain were grown in shake-flasks containing LB medium at 37 °C and shaken at 150 rpm. After 24 h cultivation, strains were transferred to a 5 L bioreactor (Baoxing, China) containing CM, aTc and M9 medium aforementioned. Air was supplied at 1.5 vvm. Agitation speed was 400 rpm, and pH value was maintained at 7.0 by addition of 5 M NaOH. The initial glucose concentration was 4 g/L. Dissolved oxygen was monitored with electrode. Fermentation broth was sampled every 3 h to examine biomass, residual glucose and metabolites. Glucose was replenished when its concentration was less than 2 g/L.

### Analytical methods

Cell concentration was measured by the 721 visible spectrophotometer (APL Instrument, Shanghai) at 600 nm with 2 mL fermentation broth added in a cuvette. To measure metabolites, fermentation broth was centrifuged at 12,000 rpm for 10 min and filtered through 0.22-μm membrane filter to remove bacteria. Residual glucose concentration was measured every 3 h by an SBA biosensor analyzer (Institute of Biology, Shandong Academy of Sciences). Aconitic acid, citric acid, alpha-ketoglutaric acid, lactic acid, and acetic acid in supernatant were analyzed by high performance liquid chromatography (HPLC) system (Shimazu, Japan) equipped with a C18 column and an SPD-20A UV detector at 210 nm. Column temperature was 25 °C. Mobile phase was 0.03% phosphoric acid at a flow rate of 0.8 mL/min. Analytical pure aconitic acid, citric acid, alpha-ketoglutaric acid, lactic acid, and acetic acid (Sigma-Aldrich, U.S.) were used as standards for quantification. F-test of two samples for variance was performed, and significance of the differences (P-values) was calculated using unpaired two-tailed *t*-tests for equal or unequal variance. All tests were performed by software GraphPad Prism 5.0.

## Supplementary information

**Additional file 1: Table S1.** Strains and vectors used in this study.

**Additional file 2: Table S2.** Oligonucleotides and primers used in this study.

**Additional file 3: Fig. S1.** Growth curve of recombinant *E. coli* harboring CRISPRi system in LB medium. Induction of CRISPRi by anhydrotetracycline (aTc) at 0, 0.125, 0.25, 0.5, 1, 2 and 4 μM..

## Data Availability

The datasets supporting the conclusions of this article are included within the article and its additional files.

## Abbreviations

CRISPRi: CRISPR interference
TCA: Tricarboxylic acid
EMP: Embden–Meyerhof–Parnas
PK: Pyruvate kinase
IDH: Isocitrate dehydrogenase
ACO: Aconitase
RT-qPCR: Reverse transcription and quantitative PCR
BPR: Base-pairing region
DH: dCas9 handle
sgRNA: Single guide RNA
asRNA: Antisense RNA
RNAi: RNA interference
sRNAs: Synthetic small-regulatory RNA
NHEJ: Non-homologous end joining
QS: Quorum sensing
aTc: Anhydrotetracycline
CM: Chloramphenicol
PEP: Phosphoenolpyruvate
BSA: Bovine serum albumin
HPLC: High performance liquid chromatography
