# Supplementary material for: CRISPR interference-guided modulation of glucose pathways to boost aconitic acid production in Escherichia coli
Source: Microb Cell Fact. 2020 Sep 3;19:174. doi: 10.1186/s12934-020-01435-9 (PMC7470443; doi:10.1186/s12934-020-01435-9)
Supplement: Supplementary file 1 — Additional file 1: Table S1. Strains and vectors used in this study. [file 12934_2020_1435_MOESM1_ESM.docx]

**Table S2 Oligonucleotides and primers used in this study.**

| **Primers** | **Sequences (5’-3’)** | **Vectors** |
| --- | --- | --- |
| **Oligonucleotides used for sgRNA synthesis** | | |
| BspQI-None-F | aaaTTCGACGTGAACTTGGTACG | pdCas9-none |
| None-BspQI-R | aacCGTACCAAGTTCACGTCGAA |  |
| BspQI-IcdA1-F | aaaCGTTGAGTTTGCCGTTTTGC | pdCas9-icdA1 and |
| IcdA1-BspQI-R | aacGCAAAACGGCAAACTCAACG | pdCas9-pykF1icdA1 |
| BspQI-IcdA2-F | aaaAGCGTCGACCACTTTCAGCA | pdCas9-icdA2 |
| IcdA2-BspQI-R | aacTGCTGAAAGTGGTCGACGCT |  |
| BspQI-IcdA3-F | aaaGATTTTACGCTCGCCTTTAT | pdCas9-icdA3 |
| IcdA3-BspQI-R | aacATAAAGGCGAGCGTAAAATC |  |
| BspQI-PykA1-F | aaaTTTTGTTCTGCGAAGCCTTC | pdCas9-pykA1 |
| PykA1-BspQI-R | aacGAAGGCTTCGCAGAACAAAA |  |
| BspQI-PykA2-F | aaaTTATTATCGCGATCTGTTGC | pdCas9-pykA2 |
| PykA2-BspQI-R | aacGCAACAGATCGCGATAATAA |  |
| BspQI-PykA3-F | aaaGCGCGCATTTTGTGATCTTC | pdCas9-pykA3 |
| PykA3-BspQI-R | aacGAAGATCACAAAATGCGCGC |  |
| BspQI-PykF1-F | aaaTCCGATGGTGCAAACAATTT | pdCas9-pykF1 and |
| PykF1-BspQI-R | aacAAATTGTTTGCACCATCGGA | pdCas9-pykF1icdA1 |
| BspQI-PykF2-F | aaaCCGGACCTTTGGTATCAAGC | pdCas9-pykF2 |
| PykF2-BspQI-R | aacGCTTGATACCAAAGGTCCGG |  |
| BspQI-PykF3-F | aaaGCCGATAACAGATTTATCAG | pdCas9-pykF3 |
| PykF3-BspQI-R | aacCTGATAAATCTGTTATCGGC |  |
| **Primers used for colony PCR** | | |
| BspQI-none-F | aaaTTCGACGTGAACTTGGTACG | pdCas9-none |
| sgRNA-CX-R | GCGGAATATATCCCTAGGCCTGCAG |  |
| BspQI-icdA1-F | aaaCGTTGAGTTTGCCGTTTTGC | pdCas9-icdA1 and |
| sgRNA-CX-R | GCGGAATATATCCCTAGGCCTGCAG | pdCas9-pykF1icdA1 |
| BspQI-icdA2-F | aaaAGCGTCGACCACTTTCAGCA | pdCas9-icdA2 |
| sgRNA-CX-R | GCGGAATATATCCCTAGGCCTGCAG |  |
| BspQI-icdA3-F | aaaGATTTTACGCTCGCCTTTAT | pdCas9-icdA3 |
| sgRNA-CX-R | GCGGAATATATCCCTAGGCCTGCAG |  |
| BspQI-pykA1-F | aaaTTTTGTTCTGCGAAGCCTTC | pdCas9-pykA1 |
| sgRNA-CX-R | GCGGAATATATCCCTAGGCCTGCAG |  |
| BspQI-pykA2-F | aaaTTATTATCGCGATCTGTTGC | pdCas9-pykA2 |
| sgRNA-CX-R | GCGGAATATATCCCTAGGCCTGCAG |  |
| BspQI-pykA3-F | aaaGCGCGCATTTTGTGATCTTC | pdCas9-pykA3 |
| sgRNA-CX-R | GCGGAATATATCCCTAGGCCTGCAG |  |
| BspQI-pykF1-F | aaaTCCGATGGTGCAAACAATTT | pdCas9-pykF1and |
| sgRNA-CX-R | GCGGAATATATCCCTAGGCCTGCAG | pdCas9-pykF1icdA1 |
| BspQI-pykF2-F | aaaCCGGACCTTTGGTATCAAGC | pdCas9-pykF2 |
| sgRNA-CX-R | GCGGAATATATCCCTAGGCCTGCAG |  |
| BspQI-pykF3-F | aaaGCCGATAACAGATTTATCAG | pdCas9-pykF3 |
| sgRNA-CX-R | GCGGAATATATCCCTAGGCCTGCAG |  |
| **Primers for DNA sequencing** | | |
| sgRNA-CX-F | TGTTTGTCGGTGAACGCTCTCTACTAG | All CRISPRi |
| sgRNA-CX-R | GCGGAATATATCCCTAGGCCTGCAG | recombinant vectors |
| **Primers for RT-qPCR** | | |
| 16S rRNA-RT-F | CCTACGGGAGGCAGCAG | - |
| 16S rRNA-RT-R | ATTACCGCGGCTGCTGG |  |
| icdA-RT-F | ACCCTGGCTCTATTATTCTCT | - |
| icdA-RT-R | ACGGTCTTCGCATTGATT |  |
| pykA-RT-F | GAACTTTTCTCACGGCTCG | - |
| pykA-RT-R | GGTGGATACACGGATTTTGG |  |
| pykF-RT-F | CAACAATGACAACCGTAAAC | - |
| pykF-RT-R | GCCCTGAGTAGCAACCAC |  |

Primers were designed by Primer Premier 5.0 software using sequences downloaded from NCBI (<http://www.ncbi.nlm.nih.gov/> ) as templates. The nucleotides labeled with gray background are restriction endonuclease sites.
