# Supplementary material for: CRISPR interference-guided modulation of glucose pathways to boost aconitic acid production in Escherichia coli
Source: Microb Cell Fact. 2020 Sep 3;19:174. doi: 10.1186/s12934-020-01435-9 (PMC7470443; doi:10.1186/s12934-020-01435-9)
Supplement: Supplementary file 2 — Additional file 2: Table S2. Oligonucleotides and primers used in this study. [file 12934_2020_1435_MOESM2_ESM.docx]

**Table S1 Strains and vectors used in this study.**

| Vectors/Strains | Description | Source |
| --- | --- | --- |
| Vectors |  |  |
| plv-dCas9-sgRNA | CRISPRi vector, chloramphenicol (CM)^R^ | (1) |
| pdCas9-none | CRISPRi vector, non-targeting sgRNA, CM^R^ | This study |
| pdCas9-icdA1 | CRISPRi vector, sgRNA targeting icdA1, CM^R^ | This study |
| pdCas9-icdA2 | CRISPRi vector, sgRNA targeting icdA2, CM^R^ | This study |
| pdCas9-icdA3 | CRISPRi vector, sgRNA targeting icdA3, CM^R^ | This study |
| pdCas9-pykA1 | CRISPRi vector, sgRNA targeting pykA1, CM^R^ | This study |
| pdCas9-pykA2 | CRISPRi vector, sgRNA targeting pykA2, CM^R^ | This study |
| pdCas9-pykA3 | CRISPRi vector, sgRNA targeting pykA3, CM^R^ | This study |
| pdCas9-pykF1 | CRISPRi vector, sgRNA targeting pykF1, CM^R^ | This study |
| pdCas9-pykF2 | CRISPRi vector, sgRNA targeting pykF2, CM^R^ | This study |
| pdCas9-pykF3 | CRISPRi vector, sgRNA targeting pykF3, CM^R^ | This study |
| pdCas9-pykF1icdA1 | CRISPRi vector, sgRNA simultaneously targeting pykF1 and sgRNA icdA1, CM^R^ | This study |
| Strains |  |  |
| *E. coli* BL21(DE3) | Wild-type strain, F^-^*omp*T *hsd*S(r_B_^-^ m_B_^-^) *gal dcm*(DE3) | Biomed |
| *E. coli* TOP10 | Competent cells | Biomed |
| *E. coli* BL21(DE3)  +pdCas9-none | Recombinant *E. coli* BL21(DE3) carrying vector pdCas9-none | This study |
| *E. coli* BL21(DE3)  +pdCas9-icdA1 | Recombinant *E. coli* BL21(DE3) carrying vector pdCas9-icdA1 | This study |
| *E. coli* BL21(DE3)  +pdCas9-icdA2 | Recombinant *E. coli* BL21(DE3) carrying vector pdCas9-icdA2 | This study |
| *E. coli* BL21(DE3)  +pdCas9-icdA3 | Recombinant *E. coli* BL21(DE3) carrying vector pdCas9-icdA3 | This study |
| *E. coli* BL21(DE3)  +pdCas9-pykA1 | Recombinant *E. coli* BL21(DE3) carrying vector pdCas9-pykA1 | This study |
| *E. coli* BL21(DE3)  +pdCas9-pykA2 | Recombinant *E. coli* BL21(DE3) carrying vector pdCas9-pykA2 | This study |
| *E. coli* BL21(DE3)  +pdCas9-pykA3 | Recombinant *E. coli* BL21(DE3) carrying vector pdCas9-pykA3 | This study |
| *E. coli* BL21(DE3)  +pdCas9-pykF1 | Recombinant *E. coli* BL21(DE3) carrying vector pdCas9-pykF1 | This study |
| *E. coli* BL21(DE3)  +pdCas9-pykF2 | Recombinant *E. coli* BL21(DE3) carrying vector pdCas9-pykF2 | This study |
| *E. coli* BL21(DE3)  +pdCas9-pykF3 | Recombinant *E. coli* BL21(DE3) carrying vector pdCas9-pykF3 | This study |
| *E. coli* BL21(DE3)  +pdCas9-pykF1icdA1 | Recombinant *E. coli* BL21(DE3) carrying vector pdCas9-pykF1icdA1 | This study |

1. Lv L, Ren YL, Chen JC, Wu Q, Chen GQ. Application of CRISPRi for prokaryotic metabolic engineering involving multiple genes, a case study: controllable P(3HB-co-4HB) biosynthesis. Metab Eng. 2015; 29:160–168. https://doi.org/10.1016/j.ymben.2015.03.013.
