# Supplementary material for: CRISPR interference-guided modulation of glucose pathways to boost aconitic acid production in Escherichia coli
Source: Microb Cell Fact. 2020 Sep 3;19:174. doi: 10.1186/s12934-020-01435-9 (PMC7470443; doi:10.1186/s12934-020-01435-9)
Supplement: Supplementary file 3 [file 12934_2020_1435_MOESM3_ESM.docx]

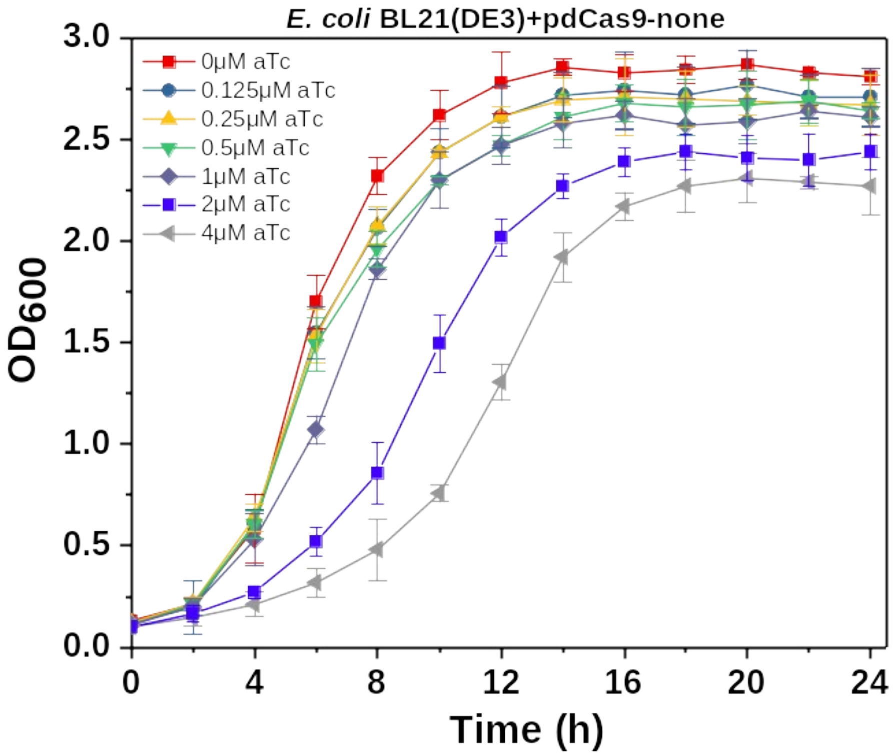

E. coli BL21(DE3)+pdCas9-none
0μM aTc
0.125μM aTc
0.25μM aTc
0.5μM aTc
1μM aTc
2μM aTc
4μM aTc
OD600
Time (h)
0.0
0.5
1.0
1.5
2.0
2.5
3.0
0
4
8
12
16
20
24
